# Relevance of mexiletine in the era of evolving antiarrhythmic therapy of ventricular arrhythmias

**DOI:** 10.1007/s00392-024-02383-9

**Published:** 2024-02-14

**Authors:** Nawar Alhourani, Julian Wolfes, Hilke Könemann, Christian Ellermann, Gerrit Frommeyer, Fatih Güner, Philipp Sebastian Lange, Florian Reinke, Julia Köbe, Lars Eckardt

**Affiliations:** Department of Cardiology II: Electrophysiology, University Hospital Münster, Münster, Germany

**Keywords:** Mexiletine, Ventricular tachyarrhythmias, Long QT syndrome, Sodium channel, Sudden cardiac death

## Abstract

Despite impressive developments in the field of ventricular arrhythmias, there is still a relevant number of patients with ventricular arrhythmias who require antiarrhythmic drug therapy and may, e.g., in otherwise drug and/or ablation refractory situations, benefit from agents known for decades, such as mexiletine. Through its capability of blocking fast sodium channels in cardiomyocytes, it has played a minor to moderate antiarrhythmic role throughout the recent decades. Nevertheless, certain patients with structural heart disease suffering from drug-refractory, i.e., mainly amiodarone refractory ventricular arrhythmias, as well as those with selected forms of congenital long QT syndrome (LQTS) may nowadays still benefit from mexiletine. Here, we outline mexiletine’s cellular and clinical electrophysiological properties. In addition, the application of mexiletine may be accompanied by various potential side effects, e.g., nausea and tremor, and is limited by several drug-drug interactions. Thus, we shed light on the current therapeutic role of mexiletine for therapy of ventricular arrhythmias and discuss clinically relevant aspects of its indications based on current evidence.

**Graphical abstract:**

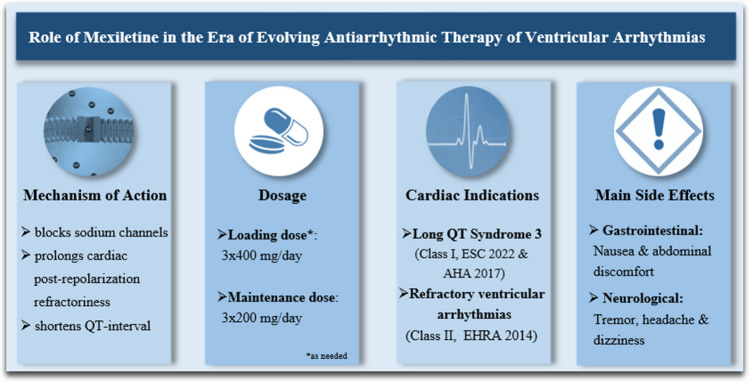

## Introduction

The management of ventricular arrhythmias has dramatically changed over the last decades. Significant achievements in defibrillator and catheter ablation therapy as well as the concern about serious drug-related side effects have resulted in a decline in antiarrhythmic drug therapy with only few evolving drug developments in the field of arrhythmias. This is reflected by a steady increase in defibrillator implantations and catheter ablations of supra- and ventricular tachyarrhythmias in patients with or without structural heart disease [1–6]. In spite of these advances, there is still a relevant number of patients with ventricular arrhythmias who require (additional) antiarrhythmic drug therapy and may benefit from drugs known for decades. One of these almost forgotten drugs is mexiletine. Mexiletine was developed by Boehringer Ingelheim (Mexitil®) in the late 1960s and showed its first positive clinical results in 1973 in a study by Talbot et al. [7], who tested it in suppressing ventricular arrhythmias mainly occurring after myocardial infarction or induced by digitalis toxicity. Afterwards, mexiletine was increasingly used as an antiarrhythmic drug and continued to prove its effectiveness with an acceptable safety profile among various groups of patients for a long period of time [8]. However, while the role of mexiletine in cardiology has gradually deteriorated, it has gained relevance in other areas such as neurology and anesthesiology. Mexiletine is, for example, nowadays considered a first-line treatment in patients with non-dystrophic myotonia and may reduce chronic neuropathic pain [8, 9].

For commercial reasons, Mexitil® was withdrawn from the European market in 2008, which tremendously restricted its medical accessibility. Although mexiletine was authorized in Europe in 2018 as Namuscla® with an orphan drug license, its use is officially limited to neurological indications [10]. Not to mention the resulting outrageous increase in this drug’s price [11], the antiarrhythmic implementation of mexiletine in Europe depends, therefore, predominantly on international imports from countries such as Japan and Canada [12]. In this review, we aim to provide insight into the pharmacological properties and associated side effects of mexiletine as well as comment on current evidence regarding its role in clinical electrophysiology.

## Clinical pharmacology of mexiletine

According to Vaughan-Williams’s classification, mexiletine belongs to class IB of antiarrhythmic drugs by blocking sodium channels in cardiomyocytes [13]. Owing to its molecular similarities to lidocaine, mexiletine is also referred to as “oral lidocaine” [14].

### Pharmacodynamic properties

**Fig. 1:**
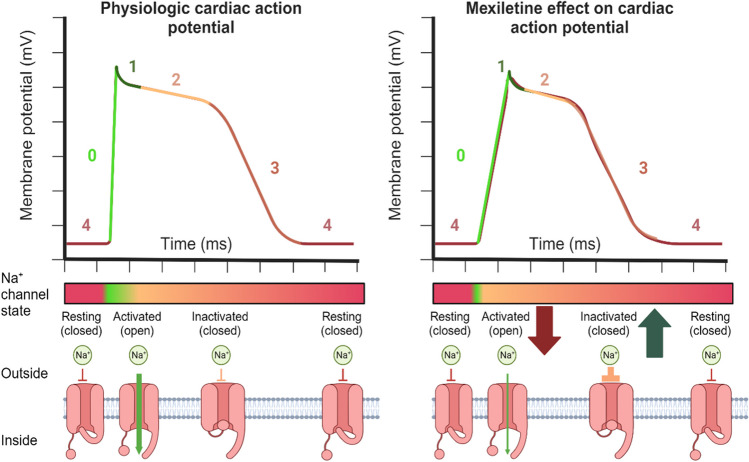
Cellular effects of mexiletine on the cardiac action potential

Mexiletine blocks fast sodium channels in different types of myocytes. The function of these voltage-gated sodium channels is essential in maintaining cardiac rhythm. During phase 0 of the cardiac action potential, sodium influx (I_Na_) into the cardiomyocytes dominates, which in turn allows the cells to reach a depolarized state. Both open and inactivated sodium channels are antagonized at a specific receptor by mexiletine (Fig. 1). This depresses, as a result, excitability and slows conduction predominantly in the ventricular cells. In comparison to other class I drugs, class IB agents detach from the blocked channels more quickly. This translates into a minimal effect at normal heart rates, whereas at faster rates the blocking effect accumulates and causes a marked reduction in the depolarization slope, i.e., positive use dependence. Additionally, the blockade of these sodium channels alters the late sodium influx (I_NaL_) during phase 2 of the cardiac action potential; this diminishes the action potential duration [15]. Cumulatively, the effective refractory period is shortened, yet the ratio of the effective refractory period to action potential duration, i.e., post-repolarization refractoriness, is prolonged [16–20]. On the other hand, mexiletine exerts a negligible effect on voltage-gated potassium channels [21].

Experimental studies have proven that ischemic tissue is more sensitive to mexiletine than normally perfused tissue: a well-known phenomenon displayed by lidocaine [14]. Burke and Berman showed, for example, that hypoxia induced an increased response to mexiletine in canine Purkinje fibers [22]. In another study by Amerini et al*.* [23] mexiletine was able to effectively suppress ventricular arrhythmias induced by reperfusion in guinea pig isolated hearts.

Regarding its influence on hemodynamic parameters, the results of the so far available clinical studies are controversial. One of these studies demonstrated that mexiletine causes, if any, only slight increases in heart rate and blood pressure. This observation could be explained by the anesthetic effect of intravenously administered mexiletine [24]. However, in the majority of these studies [12], especially those examining patients with ventricular tachyarrhythmias and ischemic heart disease and/or congestive heart failure, mexiletine altered neither the measured hemodynamic variables (heart rate and blood pressure) nor the assessed left ventricular systolic and diastolic functions (left ventricular ejection fraction, stroke volume, and end diastolic volume) [25–27]. Supporting evidence is presented in the study of Korkushko from 1987, which confirmed the lack of a significant effect on the contraction time, myocardial tension index, isometric contraction phase, and cardiac index in patients who received mexiletine for the treatment of ventricular premature contractions [28]. Yet, mexiletine was also able to exert negative inotropic effects, i.e., decrease in the cardiac index, in a small group of patients with severe heart failure [29].

### Pharmacokinetic properties

Orally administered mexiletine displays a high bioavailability, approaching 90%, as it gets almost completely absorbed from the gastrointestinal system and undergoes only a minimal first-pass hepatic metabolism. The peak plasma concentration is reached in 2–4 hours. Steady state is achieved after regular oral administration in approximately 4 to 5 days. On the other hand, intravenously administered mexiletine undergoes extensive tissue uptake. The drug’s distribution takes place in two phases: an initial rapid phase followed by a long and slow phase. Its metabolism occurs principally in the liver with an elimination half-life of 8 to 16 hours [16, 30].

## Therapeutic uses

Mexiletine demonstrated its effectiveness in various arrhythmogenic settings. Initial studies quantified its effect in terms of reduction of premature ventricular beats burden [31–35]. Later on, mexiletine was tested for the suppression of recurrent, therapy-resistant ventricular tachycardia/fibrillation [36–40]. Besides, this antiarrhythmic drug brought hope into the preventive treatment of patients with primary arrhythmia syndromes and specific genetic mutations.

### Premature ventricular contractions (PVC)

Using pre-post designed historic studies as well as controlled trials, mexiletine was assessed for its ability to suppress PVCs. In a study conducted by Duke et al. [41] in 1988 on 100 patients with non-sustained ventricular arrhythmias, the PVC frequency was significantly reduced after 3 months of receiving mexiletine at a daily dosage between 600 and 900 mg. The target reduction was defined as ≥ 70% suppression of the PVC rate. This effect was maintained over a long-term follow-up period of 12 months. As presented in Table 1, mexiletine succeeded in almost all the presented trials in proving its antiarrhythmic effect in controlling PVCs [31–35]. Of note, there is nowadays barely any indication for mexiletine in this situation left. Symptomatic idiopathic PVCs as well as those in patients with structural heart disease are mainly managed by catheter ablation or alternatively by other antiarrhythmic drugs, e.g., class IC drugs (flecainide or propafenone) or class III drugs (amiodarone or sotalol) [42–44].

### Ventricular tachycardia/fibrillation (VT/VF)

Numerous observational studies have explored the efficacy of mexiletine in patients with recurrent VT/VF through programmed electrical stimulation. For example, in a clinical study by Whitford et al*.* [45] in 1988, one-third of the 159 included patients, who presented with refractory ventricular arrhythmias to class IA antiarrhythmic drugs, responded to either monotherapy of mexiletine or its combination with class IA drugs (quinidine, disopyramide, or procainamide). A positive response was specified by failure of VT-inducibility using programmed electrical stimulation. Other investigations analyzed mexiletine’s impact by measuring the reduction in spontaneous occurrence of ventricular arrhythmias or related implantable cardioverter-defibrillator device (ICD) therapy burden in those with arrhythmias resistant to conventional treatment (mostly amiodarone). In Table 2, the results of some of the most relevant, mainly historic, studies are summarized [36–40]. Similar to the presented PVC data, today’s therapeutic role of mexiletine for recurrent VT/VF is limited. Patients with sustained ventricular tachycardia in the presence of structural heart disease predominately receive ICD therapy for secondary prevention, often receive additional class III antiarrhythmic drug therapy and/or undergo catheter ablation. This is supported by the large randomized VANISH trial [38, 46], which demonstrated inferiority of escalation of antiarrhythmic drug therapy (amiodarone without or with mexiletine) to catheter ablation therapy in patients with ischemic heart disease and recurrent ICD therapy.

### Long QT syndrome (LQTS)

Congenital LQTS is a genetic ion channelopathy distinguished by QT interval prolongation and T-wave abnormalities, which represents one of the primary arrhythmia syndromes with a pronounced risk of life-threatening ventricular arrhythmias and sudden cardiac death. According to the underlying mutations, LQTS is further classified into three main genetic subtypes: LQTS 1, LQTS 2, and LQTS 3. The associated genes are KCNQ1, KCNH2, and SCN5A, respectively [47–49]. Mexiletine’s capability of shortening the QT interval made its applicability in treating patients with LQTS appealing. In experimental heart models, mexiletine significantly reduced the QT interval and remarkably prevented polymorphic ventricular tachycardia after a pharmacological induction of QT interval prolongation [50, 51]. Furthermore, clinical trials confirmed its effectiveness in patients. Mazzanti et al. [52] illustrated in a retrospective cohort study the relevant shortening of the QT interval and subsequent decline in cardiac events in patients diagnosed with LQTS 3 who received mexiletine. Although SCN5A mutations mainly result in gain of function, which translates into increased sodium inflow and in turn explains the success of mexiletine therapy, heterogeneous reactions to this therapy in patients with LQTS 3 were observed. Diverse evidence determined an association with specific mutations of the SCN5A gene, i.e., mexiletine-sensitive mutations [53]. Mexiletine became especially attractive in those with bradycardia-induced QT interval prolongation, as the mainstay treatment option with nonselective beta-blockers can actually be aggravating [54]. In particular in patients with LQTS 3, where arrhythmogenic triggers are known to be sleep and long pauses, the role of beta-blocker remains controversial and uncertain [55]. A case report of a LQTS 3 patient illustrated that replacing propranolol with mexiletine diminished the occurrence of torsade de pointes [56]. Interestingly, a recent experimental study revealed a further potential therapeutic aspect of mexiletine in the treatment of SCN5A overlap syndrome, where in addition to the LQTS 3 typical increase in late sodium influx, a decrease in the peak sodium currents typical of Brugada syndrome/chronic conduction disease is evident. On genetically altered cardiomyocytes expressing SCN5A overlap syndrome phenotype, mexiletine acutely decreased the late sodium current (I_NaL_) and chronically increased the sodium current (I_Na_) peak, promoting it to eventually become a therapeutic option for this complex genetic syndrome [57]. Mexiletine’s efficacy is not only limited to patients with diagnosed LQTS 3, but may also be beneficial in patients with potassium-mediated LQTS 2 [58]. In view of the results of a meta-analysis evaluating sodium channel blockers in all LQTS types, mexiletine showed more significant QT_c_ interval shortening in both LQTS 3 and LQTS 2 in comparison with flecainide and ranolazine [59]. The therapeutic scope of mexiletine may further extend to include drug-induced LQTS [50]. Johannesen et al*.* [60] nicely demonstrated mexiletine’s effect of QT_c_ interval shortening in patients pre-exposed to dofetilide. This beneficial interaction may reflect the counteracting block of late sodium current exhibited by mexiletine to dofetilide’s potassium channel blocking effect. Badri et al*.* [61] also favorably established the utilization of mexiletine in a small group of patients who failed to respond to conventional therapy for acquired LQTS.

## Dosage and administration

Owing to the narrow therapeutic window of mexiletine, the transition point between efficacy and toxicity is important. To reach a steady-state plasma concentration between 0.75 and 2.0 mg/l, the initial maintenance dose is 600 mg/day (i.e., 200 mg capsules, three times a day). In case of insufficient effect, the daily dose can be increased to 900 mg with a maximum of 1200 mg [16]. Multiple studies proved that administration of a total amount of 900 mg/day resulted in a significant reduction in the targeted arrhythmia. In historic investigations, Podrid and Lown showed that the majority of patients with premature ventricular contractions responded to the treatment regimen of mexiletine at a daily dose of 600–900 mg [62].

A loading dose of orally administered 400 mg followed by 200 mg in 2 hours and then on an 8-hourly basis may be used to quickly establish a therapeutic peak plasma concentration [16]. Alternatively, a starting dose of 400 mg three times a day for 2–3 days is sufficient. In acute situations or inapplicability of oral administration, mexiletine can be administered intravenously. This can be initiated with 125–250 mg over 10–20 min followed by an infusion of 250 mg or 500 mg over 1 hour and 3.5 hours, respectively [16]. The daily maintenance dose of 600–900 mg/24 h as a continuous infusion is equivalent to the oral dosage.

Because of its dominant hepatic metabolism, a dosage adjustment is required in patients with hepatic insufficiency. If liver cirrhosis is present, a relevant reduction to 25–30% of the usual maintenance dose is recommended [63], whereas in case of renal impairment, no dosage adjustment is needed [16].

Few studies have investigated mexiletine in pregnant and breastfeeding patients. Mexiletine is lipid-soluble, which enables its passage across the placenta and excretion in breast milk. Reported complications included fetal bradycardia, neonatal failure to thrive, and feeding incompetence [64–66]. Due to the lack of extensive studies and experience, the application of mexiletine should be avoided in pregnant and breastfeeding patients, according to the producing pharmaceutical company [67].

## Adverse effects, drug-drug interactions, and contraindications

### Adverse effects

Upon using mexiletine, diverse side effects, mostly related to the gastrointestinal tract and nervous system, may occur. A recent systematic review showed that gastrointestinal adverse effects with a predominance of nausea and abdominal pain/discomfort occurred in 33% of the included patients [12]. Similarly, 31% of the studied patients suffered from neurological adverse events. Tremors, followed by headaches and dizziness, were the most prevalent symptoms [12]. Notably, the IMPACT study [68] displayed tremor to be the only significantly reported adverse effect in patients receiving mexiletine in comparison to placebo. Problems with coordination and paresthesia were also reported, and psychiatric complications including insomnia and depression were mentioned [12]. Less commonly, hematological side effects, particularly thrombocytopenia, were published [69]. Cardiac side effects including sinus bradycardia, heart failure, and cardiac chest pain were also reported, but do not seem to be of major relevance for most patients [12]. As it shortens the QT_c_ interval, ventricular proarrhythmia (i.e., polymorphic VT of the torsade de pointes type) is neglectable. In contrast to class IC agents, mexiletine does not induce relevant proarrhythmia (i.e., monomorphic VT) in patients with structural heart disease, illustrating a rather safe cardiovascular risk profile. Based on the results of the landmark cardiac arrhythmia suppression trial (CAST) [70], most class I antiarrhythmic drugs, although only class IC drugs were tested in the trial, were subsequently labeled proarrhythmogenic with potentially fatal sequelae in patients with structural heart disease [71]. Nevertheless, multiple double-blinded studies failed to prove a statistically significant increase in proarrhythmia or mortality in patients with acute myocardial infarction who received mexiletine [68, 72, 73].

To minimize the potential adverse effects related to mexiletine, it is recommended to ingest the capsules with food. Furthermore, the addition of beta-blockers to mexiletine can significantly reduce accompanying tremors [74]. The incidence of adverse events caused by mexiletine appears to be dose-related [68]. Based on this observation, combining mexiletine with other antiarrhythmic drugs was tested. The combination with quinidine led to an actual reduction in the required effective dose of mexiletine and thus the incidence of related adverse effects [75].

### Drug-drug interactions and contraindications

The absorption of orally administered mexiletine can be induced through metoclopramide, whereas cimetidine and antacids decelerate its gastrointestinal absorption. Since the elimination of mexiletine hugely depends on hepatic enzymes, the interaction with some drugs known as “enzyme inducers or inhibitors” is predictable. Co-administration of rifampin, phenytoin, and phenobarbital reduces mexiletine’s plasma levels. However, chloramphenicol and isoniazid inhibit its metabolism [30]. Also, mexiletine can positively influence theophylline, in that it remarkably increases theophylline’s plasma levels. To avoid theophylline toxicity, it is essential to measure theophylline’s plasma levels and adjust mexiletine’s dosage accordingly [16].

In patients with recurrent and/or incessant VT, the combination of mexiletine with class III antiarrhythmic drugs remains a relevant drug combination regimen. Amiodarone is known to increase the plasma levels of class I antiarrhythmic drugs due to its potent CYP450 inhibition [76]. This fact may contribute to the enhanced antiarrhythmic effect in suppressing ventricular arrhythmias when amiodarone is added to mexiletine [77]. In an experimental investigation, the combination of mexiletine with sotalol resulted in a significant reduction in QT_c_ interval prolongation and, in turn, the incidence of torsade de pointes in comparison to sotalol alone [78].

Hypersensitivity to mexiletine, untreated high degree second or third atrioventricular block, and sinus node dysfunction are contraindications for mexiletine. Special caution should be taken in the presence of mild to moderate hepatic impairment. Electrolyte abnormalities should be corrected to decrease the incidence of possibly associated side effects [67]. Remarkably, ventricular tachyarrhythmias, coronary artery disease, and heart failure are also listed as contraindications in the official information leaflet of Namuscla® [67].

## Conclusion

**Fig. 2:**
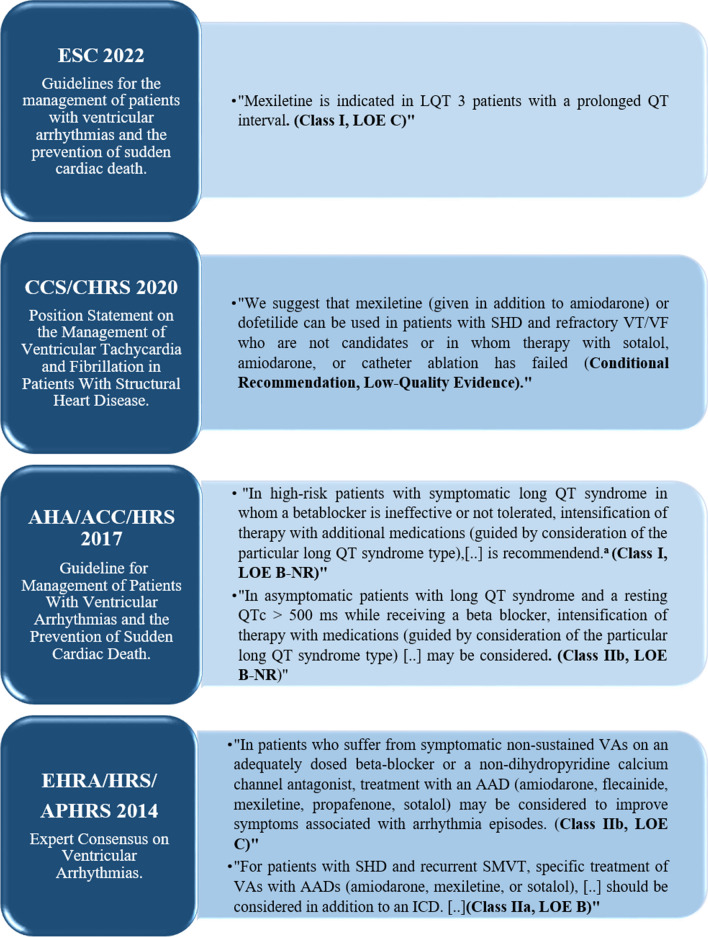
Role of mexiletine in the recent international guidelines [42, 79–81]. *LOE* level of evidence, *SHD* structural heart disease; *VA* ventricular arrhythmias, *SMVT* sustained monomorphic ventricular tachycardia, *AAD* antiarrhythmic drugs. ^a^High-risk patients with long QT syndrome include those with QT_c_ > 500 ms, genotypes long QT syndrome type 2 and long QT syndrome type 3, females with genotype long QT syndrome type 2, < 40 years of age, onset of symptoms at < 10 years of age, and patients with prior cardiac arrest or recurrent syncope

The role of mexiletine in the management of ventricular arrhythmias, which is reflected in the international cardiovascular guidelines, has changed over the years [42, 79–81] (Fig. 2). Mexiletine plays a fading role in pharmacological suppression of premature ventricular contractions and exhibits inferiority to catheter ablation in management of recurrent ventricular arrhythmias in patients with structural heart disease. In the era of modern antiarrhythmic therapy, mexiletine remains one of the individualized antiarrhythmic options applied to patients with recurrent drug- and ablation-refractory ventricular arrhythmias or those with LQTS 3. In the context of managing recurrent ventricular tachyarrhythmias, it is mainly used in combination with amiodarone or sotalol or if the latter are not sufficient or tolerated as a stand-alone therapy. A maintenance daily dose of 600–900 mg is recommended. Its associated side effects are usually tolerable but should be considered. As mexiletine exhibits a niche indication in cardiac electrophysiology, we propose additional prospective data to further define the target patient collectives that would most likely profit from the treatment.

## Appendix

Figure 1, Tables 1, 2, Figure 2

**Table 1:** Comparison of the results of historical studies on the effect of mexiletine on PVC suppression [31–35]

Study’s authorship (year of publication)	No. of included patients	Duration of treatment	Drug/daily dosage	% of PVC reduction
Placebo-—controlled crossover studies				
Masotti et al. (1984)	26	3 weeks	Mexiletine/600–1200 mg	≥ 50% in 79% (19/24 patients)
Poggesi et al. (1989)	144	5 weeks	Mexiletine/600 mg	≥ 75% in 82% (104/127 patients)
Fracalossi et al. (1989)	14	1 week	Mexiletine/800 mg	≥ 90% in 29% (4/14 patients)
Active treatment—controlled crossover trial				
Sakurada et al. (1991)	75	8 weeks	Mexiletine/300–600 mg vs. disopyramide/300–600 mg vs. combination therapy/300 + 300 mg	≥ 75% in 49% (18/37 patients) vs ≥ 75% in 47% (18/35 patients) vs ≥ 75% in 32% (6/19 patients)
Comparative parallel studies				
Morganroth et al. (1987)	246 vs. 245	12 weeks	Mexiletine/600–1200 mg vs. quinidine/800–1600 mg	≥ 70% in 31% (71/232) vs ≥ 70% in 32% (73/225)

**Table 2:** Comparison of the results of studies on the effect of mexiletine on VT/VF suppression [36–40]

Study’s authorship (year of publication)	No. of included patients	Period of follow-up	Mexiletine’s dose/day	ICD therapy/patient before vs. on mexiletine	VT/VF episodes/patient before vs. on mexiletine
Mugnai et al. (2021)	34	~ 26.5 months	400–600 mg	~ 3.41 vs. 1.53 *p* = 0.02	~ 2.18 vs. 0.97 *p* = 0.002
Deyell et al. (2018) VANISH	19 (11 in mexiletine arm and 8 in ablation arm)	9–30 months	200–900 mg	~ 0.72 on mexiletine vs. 0.38 after ablation *p* = 0.07	~ 0.64 on mexiletine vs. 0.25 after ablation (electrical storms) *p* = 0.01
Sobeich et al. (2017)	17	~ 13.9 months	400–600 mg	~ 18.6 vs. 0.53 *p* < 0.001	~ 16.76 vs. 3.89 *p* = 0.01
Gao et al. (2013)	29	~ 12 months	300 mg	**Short term (3 months):** 0.06 vs. 0 *p* = 0.003 **Long term (12 months):** 0.14 vs. 0.03 *p* = 0.084	**Short term (3 months):** 0.41 vs. 0.07 *p* = 0.001 **Long term (12 months):** 0.59 vs. 0.31 *p* = 0.053
